# Effect of Interactions Between Endothelial Lipase Gene Polymorphisms and Traditional Cardiovascular Risk Factors on Coronary Heart Disease Susceptibility

**DOI:** 10.31083/RCM37356

**Published:** 2025-07-25

**Authors:** Chunhui He, Xingming Song, Ting He, Qing Tian, Yuhui Zhang, Halisha Airikenjiang, Dilihumaer Abulaiti, Haitang Qiu, Mengbo Zhu, Juan Yang, Jian Zhang, Ying Gao

**Affiliations:** ^1^Department of Cardiology, Beijing Anzhen Hospital, Capital Medical University, National Clinical Research Center for Cardiovascular Diseases, 100029 Beijing, China; ^2^Department of Geriatric, Huangshi Central Hospital, Affiliated Hospital of Hubei Polytechnic University, Edong Healthcare Group, 435000 Huangshi, Hubei, China; ^3^Beijing Key Laboratory of Preclinical Research and Evaluation for Cardiovascular Implant Materials, Animal Experimental Centre, Fuwai Hospital, National Centre for Cardiovascular Disease, Chinese Academy of Medical Sciences and Peking Union Medical College, 100037 Beijing, China; ^4^Heart Failure Care Unit (HFCU), Heart Failure Center, Fuwai Hospital, National Center for Cardiovascular Disease, Chinese Academy of Medical Sciences and Peking Union Medical College, 100037 Beijing, China; ^5^Department of Comprehensive Internal Medicine, First Affiliated Hospital of Xinjiang Medical University, 830011 Urumqi, Xinjiang, China; ^6^Key Laboratory of Clinical Research for Cardiovascular Medications, National Health Committee, 100037 Beijing, China

**Keywords:** gene-environment interaction, gene polymorphisms, endothelial lipase, coronary heart disease, traditional cardiovascular risk factors

## Abstract

**Background:**

Coronary heart disease (CHD) arises from a complex interplay of genetic and environmental factors. This study examines the influence of *endothelial lipase* gene polymorphisms (*rs2000813* and *rs3813082*) and their interactions with traditional cardiovascular risk factors on CHD susceptibility.

**Methods:**

This retrospective case–control study enrolled 900 CHD patients and 900 control subjects. We evaluated associations between conventional cardiovascular risk factors and polymorphisms at the *rs2000813* and *rs3813082* loci in the *endothelial lipase* gene. Multifactorial analysis was used to assess interactions between traditional risk factors and these polymorphisms. Additionally, we developed a predictive model integrating genetic variants and clinical variables to estimate CHD risk.

**Results:**

No significant differences were observed in the distribution of *rs2000813* genotypes (*CC*, *CT*, *TT*) and alleles (*C*, *T*), or *rs3813082* genotypes (*AA*, *AC*, *CC*) and alleles (*A*, *C*) between CHD and control groups, including among males. However, in females with CHD, the *rs2000813CT* genotype was significantly more frequent (49.30%) than in controls (37.80%), whereas the *CC* genotype was less frequent in the CHD group (45.00%) than in controls (55.20%). Multivariate logistic regression identified the *rs2000813CT* genotype, hypertension, ages ≥60 years, body mass index (BMI) values ≥28 kg/m^2^, total cholesterol (TC) ≥6.2 mmol/L, and apolipoprotein B (ApoB) ≥1.1 g/L as potential risk factors for CHD in women (*p* < 0.05). Gene–environment interaction analysis revealed that BMI exerted the greatest influence (12.62%). A predictive model incorporating *rs2000813* genotypes estimated CHD risk in women with an area under the curve (AUC) of 0.804.

**Conclusions:**

The *rs2000813CT* *endothelial lipase* genotype is potentially associated with an increased CHD risk in females, whereas the *CC* genotype may confer a protective effect. Integrating *endothelial lipase* gene variants with traditional cardiovascular risk factors enhances CHD risk prediction in women. Synergistic interaction between *endothelial lipase* polymorphisms and environmental factors appears to influence CHD occurrence in this population.

## 1. Introduction

Coronary heart disease (CHD) encompasses a spectrum of cardiovascular disorders resulting from myocardial ischemia, hypoxia, or necrosis due to coronary artery stenosis or occlusion. It is characterized by high morbidity and mortality rates. According to the World Health Organization’s Global Health Estimates report from December 2020, CHD remains the leading cause of death worldwide, accounting for 16% of all fatalities [1]. In China, the prevalence of CHD is increasing. The China Health Statistics Yearbook 2019 reports that in 2018, the CHD mortality rate was 120.18 per 100,000 in urban populations and 128.24 per 100,000 in rural population [2].

As a chronic condition, CHD is manageable but incurable, requiring long-term diagnosis and treatment. Its etiology primarily stems from a combination of factors, including dyslipidemia, coronary artery endothelial dysfunction, and chronic inflammation [3, 4]. Recent studies have underscored the critical role of genetic variants in CHD and pathophysiology [5, 6]. Despite the implementation of clinical interventions, such as preventive and therapeutic medications, along with guidelines for managing lipid metabolism disorders and chronic inflammatory, the prevalence of CHD remains high [7]. Elucidating the regulatory mechanisms of gene variants in lipid metabolism, inflammatory responses, and vascular endothelial injury is essential to address the underlying causes of CHD and may pave the way for significant advances in its prevention and treatment [8].

Endothelial lipase (EL), encoded by the *endothelial lipase* gene, is primarily synthesized by vascular endothelial cells. Its expression is upregulated in response to acute inflammatory stimuli, endotoxin exposure, and alterations in vascular wall shear stress. Hirata *et al*. [9] identified the *endothelial lipase* gene on the long arm of chromosome 18 (*18q21.1*), comprising 10 exons and 9 introns. EL plays a pivotal role in lipid metabolism and is implicated in metabolic syndromes, including inflammation and atherosclerosis [10]. This study investigates the effects of *endothelial lipase* polymorphisms *rs2000813* and *rs3813082*, alongside traditional cardiovascular risk factors and their interactions, on susceptibility to CHD. Furthermore, it aims to develop a risk-prediction model integrating genetic and conventional factors. This model seeks to identify high-risk individuals for early intervention, enhance the prognosis of cardiac events, and provide a theoretical foundation for novel approaches to the diagnosis, treatment, and prevention of CHD.

## 2. Materials and Methods

### 2.1 Sample Size Estimation

This study employed a case-control design. The sample size was calculated using the following parameters: an odds ratio (OR) of 1, a significance level (α) of 0.05, a power (1 - β) of 0.9, and minor allele frequencies for the *rs2000813 T* allele of 0.22 (PMAF1) and 0.30 (PMAF2), sourced from the National Center for Biotechnology Information (NCBI) database. Using the two-proportion formula in PASS software (version 21.0.3, Kaysville, UT, USA), we estimated a required sample size of 1260 participants. To account for a 20% attrition rate, an additional 252 participants were included, yielding a total sample size of 1512. This was evenly divided into 756 participants in the coronary heart disease group and 756 in the control group.

### 2.2 Study Participants

Between January 2019 and December 2021, we enrolled 900 patients diagnosed with coronary artery disease (CAD) who underwent coronary angiography or percutaneous coronary intervention (PCI) at the First Affiliated Hospital of Xinjiang Medical University as the case group. Concurrently, 900 patients who did not meet the diagnostic criteria, as confirmed by coronary angiography or coronary computed tomography angiography (CTA) during the same period, were selected as the control group. Subsequently, 720 participants from each group were randomly allocated in an 8:2 ratio to form the modeling subset, with the remaining 180 participants from each group assigned to the validation subset.

### 2.3 Case group Inclusion and Exclusion Criteria

Inclusion criteria: (1) Patients aged ≥18 years. (2) Patients hospitalized at the First Affiliated Hospital of Xinjiang Medical University between January 2019 and December 2021 with typical angina symptoms or non-invasive evidence of myocardial ischemia, a prerequisite for admission at this institution. (3) CHD diagnosis confirmed by coronary angiography, demonstrating coronary artery stenosis >50% in diameter.

Exclusion criteria: Patients were excluded if they met any of the following conditions: (1) Incomplete clinical data. (2) Presence of severe heart failure, cardiogenic shock, malignant arrhythmias, tumors, autoimmune diseases, or conditions associated with aortic stenosis. (3) Refusal to participate or noncompliance with the study protocol.

### 2.4 Control Group Inclusion and Exclusion Criteria

Inclusion criteria: (1) Participants aged ≥18 years. (2) Patients hospitalized at the First Affiliated Hospital of Xinjiang Medical University between January 2019 and December 2021 with typical angina symptoms or noninvasive evidence of myocardial ischemia, a prerequisite for admission at this institution. (3) Absence of CHD confirmed by coronary angiography or CTA.

Exclusion criteria: Patients were excluded if they met any of the following: (1) Incomplete clinical data. (2) Presence of severe heart failure, cardiogenic shock, malignant arrhythmias, tumors, or autoimmune diseases. (3) Refusal to participate or noncompliance with the study protocol.

### 2.5 Diagnostic Criteria

(1) CHD: Diagnosis required typical angina symptoms and/or non-invasive evidence of myocardial ischemia, coupled with coronary angiography demonstrating ≥50% stenosis in the main trunk, left anterior descending branch (including the diagonal branch), left circumflex artery (including the marginal branch), or right coronary artery (including the posterior descending and posterolateral branch) [11, 12].

(2) Hypertension: Defined as systolic blood pressure ≥140 mmHg and/or diastolic blood pressure of ≥90 mmHg, measured at rest on at least two separate days, or a documented history of hypertension [13].

(3) Type 2 diabetes mellitus: Diagnosed based on random blood glucose ≥11.1 mmol/L (200 mg/dL), fasting glucose levels ≥7.0 mmol/L (126 mg/dL), 2-hour post-load glucose ≥11.1 mmol/L (200 mg/dL) on an oral glucose tolerance test, or a confirmed history of type 2 diabetes mellitus [14].

(4) Smoking calculator: A pack year is defined as twenty cigarettes smoked everyday for one year [15].

(5) Alcohol consumption: Defined as ethanol intake ≥28 g/d for men or ≥14 g/d for women, calculated as ethanol (g) = alcohol volume (mL) × ethanol percentage (%) × 0.8 [16].

### 2.6 General Data Collection

Baseline data, including sex, age, body mass index (BMI), smoking history, alcohol consumption, hypertension, and type 2 diabetes mellitus, were collected from all participants.

### 2.7 Determination of Biochemical Indicators

After fasting for >12 hours, venous blood was collected from participants in the early morning into anticoagulant tubes. Serum samples were analyzed for the following indices: triglyceride (TG), total cholesterol (TC), high-density protein cholesterol (HDL-C), low-density protein cholesterol (LDL-C), apolipoproteins A and B (ApoA and ApoB), lipoprotein a (Lp(a)), serum uric acid (SUA), serum creatinine (Scr), white blood cell (WBC), neutrophil count (NE), platelet count, creatine kinase-MB (CK-MB), and cardiac troponins I and T (cTnI and cTnT). These measurements were performed by the Laboratory Department of the First Affiliated Hospital of Xinjiang Medical University using an automated biochemical analyzer (Olympus AU1000/2700, SC, USA).

### 2.8 DNA Extraction and SNPscnTM High Flux

Upon hospital admission, 3 mL of venous blood was collected from the antecubital vein of each participant and anticoagulated with 2% ethylenediaminetetraacetic acid (EDTA) disodium salt. Leukocytes were isolated by centrifugation, and genomic DNA was extracted using the phenol-chloroform method, then stored at –80 °C. The SNPscnTM high-throughput assay was employed to genotype the *endothelial lipase* gene loci *rs2000813* and *rs3813082*. Based on nucleotide variations, the *rs2000813* locus was classified into *CC*, *CT*, and *TT* genotypes, and *rs3813082* locus into *AA*, *AC*, and *CC* genotypes (**Supplementary Table 1**).

### 2.9 Statistical Methods

Statistical analyses were conducted using SPSS software (version 26.0, IBM Corp., Armonk, NY, USA). Categorical data were reported as counts and percentages (n [%]) and compared using the chi-square (χ^2^) test. For continuous data, the Kolmogorov-Smirnov test was employed to assess normality. Normally distributed continuous data were presented as means ± standard deviations and analyzed with independent-sample *t*-test for two-group comparisons or one-way analysis of variance (ANOVA) followed by the least significant difference (LSD) *t*-test for multiple-group comparisons. Non-normally distributed continuous data were expressed as medians with the 25th and 75th percentiles (M [P_25_, P_75_]) and evaluated using the Mann-Whitney U test for two groups or the Kruskal-Wallis H test for multiple groups. Spearman correlation, univariate, and multivariate logistic regression analyses were performed to identify risk factors of CHD in women. Gene-environment interactions between the endothelial lipase gene and CHD risk factors in women were assessed using multifactor dimensionality reduction (MDR) software (version 3.0.2, Computational Genetics Laboratory, Hanover, NH, USA). Receiver operating characteristic (ROC) curves were generated with GraphPad Prism (version 8.0.2, GraphPad Software, Inc., San Diego, CA, USA) to evaluate the predictive performance of the model for CHD risk in women. Statistically significance was defined as *p* < 0.05.

## 3. Results

### 3.1 Comparison of Baseline Characteristics Between Case and Control Groups

Baseline characteristics (Table 1) revealed no significant differences between the case and control groups in sex distribution, age, alcohol consumption, TG levels, ApoA, SUA, Scr, or other variables. However, the case group exhibited significantly higher prevalence rates of smoking (44.70% vs. 36.60%, *p* = 0.001), hypertension (48.60% vs. 43.10%, *p* = 0.023) and diabetes (26.20% vs. 11.90%, *p* < 0.001) compared to the control group. Additionally, the case group had higher BMI, TC, LDL-C, Lp(a), WBC, NE, platelet (PLT), CK-MB, cTnI and cTnT levels, with specific differences noted for HDL-C (1.00 ± 0.29 vs. 1.08 ± 0.32, *p* < 0.001), and ApoB (0.87 [0.73, 1.02] vs. 0.84 [0.68, 0.98], *p* < 0.001). Conversely, HDL-C and ApoB levels were significantly lower in the case group.

**Table 1.** **Comparison of baseline data between the two groups**.

Variable	Control group (n = 900)	Case group (n = 900)	χ^2^/*t*/Z	*p*-value
Male, n (%)	589 (66.40)	612 (68.00)	1.324^a^	0.271
Age, years	55.75 ± 9.17	55.98 ± 10.18	–0.511^b^	0.609
Smoking history, n (%)	329 (36.60)	402 (44.70)	12.275^a^	0.001
History of drinking, n (%)	268 (29.80)	265 (29.40)	0.024^a^	0.918
Hypertension, n (%)	388 (43.10)	437 (48.60)	5.373^a^	0.023
Diabetes, n (%)	107 (11.90)	236 (26.20)	59.937^a^	<0.001
BMI, kg/m^2^	25.40 (23.44, 29.36)	28.03 (25.77, 31.25)	–11.307^c^	<0.001
TG, mmol/L	1.66 (1.17, 2.26)	1.66 (1.19, 2.40)	–0.845^c^	0.398
TC, mmol/L	4.33 (3.89, 4.78)	4.44 (4.01, 5.10)	–4.713^c^	<0.001
HDL-C, mmol/L	1.08 ± 0.32	1.00 ± 0.29	5.037^b^	<0.001
LDL-C, mmol/L	2.73 (2.51, 3.05)	2.75 (2.60, 3.31)	–4.248^c^	<0.001
ApoA, mmol/L	1.17 (1.06, 1.30)	1.17 (1.01, 1.30)	–1.761^c^	<0.078
ApoB, mmol/L	0.84 (0.68, 0.98)	0.87 (0.73, 1.02)	–3.614^c^	<0.001
Lp(a), mmol/L	153.91 (110.88, 215.00)	176.65 (123.16, 265.80)	–4.775^c^	<0.001
SUA, µmol/L	311.61 ± 86.59	316.44 ± 91.61	–1.150^b^	0.250
Scr, µmol/L	71.00 (59.09, 82.00)	71.01 (61.00, 83.00)	–1.195^c^	0.232
WBC, ×10^9^/L	6.68 (5.53, 7.52)	8.61 (6.97, 11.06)	–17.829^c^	<0.001
NE, ×10^9^/L	3.82 (2.98, 4.60)	5.66 (4.07, 8.41)	–18.466^c^	<0.001
PLT, ×10^9^/L	216.00 (182.00, 250.00)	223.50 (191.00, 269.75)	–5.328^c^	<0.001
CK-MB, µg/L	13.60 (10.10, 16.30)	18.33 (12.47, 38.37)	–14.625^c^	<0.001
cTnI, µg/L	0.00 (0.00, 0.00)	0.00 (0.00, 0.02)	–6.531^c^	<0.001
cTnT, µg/L	0.00 (0.00, 0.03)	0.02 (0.00, 0.27)	–20.866^c^	<0.001

BMI, body mass index; TG, triglyceride; TC, total cholesterol; HDL-C, high-density lipoprotein cholesterol; LDL-C, low-density lipoprotein cholesterol; ApoA, apolipoprotein A; ApoB, apolipoprotein B; Lp(a), lipoprotein (a); SUA, serum uric acid; Scr, serum creatinine; WBC, white blood cell; NE, neutrophil count; PLT, platelet; CK-MB, creatine kinase-MB; cTnI, cardiac troponin I; cTnT, cardiac troponin T; ^a^: χ^2^, ^b^: *t*, ^c^: *Z*.

### 3.2 Association of Endothelial Lipase Gene Polymorphisms With Coronary Heart Disease Risk

**Supplementary Table 2 **presents the Hardy-Weinberg equilibrium (HWE) test results. Genotype distributions for both the CHD and control groups conformed to HWE, indicating that the study population represents a Mendelian population in genetic equilibrium. This finding supports the genetic representativeness the sample, a critical factor for the validity of genetic association studies. Additionally, haplotype linkage disequilibrium (LD) analysis of the *rs2000813* and *rs3813082* loci in the *endothelial lipase* gene was performed using the SHEsis online platform. The results yielded a D’ value of 0.158 and an r^2^ value of 0.001, suggesting no significant LD between these loci. Thus, *rs2000813* and *rs3813082* are genetically independent, an important consideration for evaluating their individual and combined effects on CHD susceptibility.

**Supplementary Table 3** summarizes the distribution of endothelial lipase gene polymorphisms between the CHD and control groups. No significant differences were observed in the distribution of *rs2000813* genotypes (*CC*, *CT*, *TT*) and alleles (*C*, *T*), or in *rs3813082* genotypes (*AA*, *AC*, *CC*) and alleles (*A*, *C*) between the groups (all *p* > 0.05). These findings suggest that, when considered independently, these polymorphisms do not significantly distinguish the CHD group from the control group in this study.

**Supplementary Table 4** details the distribution of *endothelial lipase* genes polymorphisms in male participants, comparing the CHD and control groups. No significant differences were observed in the distribution of *rs2000813* genotypes (*CC*, *CT*, *TT*) and alleles (*C*, *T*), or *rs3813082* genotypes (*AA*, *AC*, *CC*) and alleles (*A*, *C*) between the CHD and the control groups among men (all *p* > 0.05). This suggests that these polymorphisms are similarly distributed in both groups within the male population. Table 2 presents the distribution of *endothelial lipase* genes polymorphisms in female participants. In women, the *rs2000813 CT* genotype was significantly more prevalent in the CHD group than in the control group (49.30% vs. 37.80%, *p* = 0.040), whereas the *CC* genotype was less frequent (45.00% vs. 55.20%, *p* < 0.05). No significant differences were found in the distribution of the *TT* genotype or *C* and *T* alleles between the groups. For *rs3813082*, the distribution of genotypes (*AA*, *AC*, *CC*) and alleles (*A* and *C*) showed no significant differences between the CHD and control groups in women (all *p* > 0.05).

**Table 2.** **Risk analysis of coronary heart disease among different genotypes in female**.

SNP	Genotype	Control group (n = 241)	Case group (n = 229)	χ ^2^	*p*-value
rs2000813	CC	133 (55.20)	103 (45.00)	6.417	0.040
	CT	91 (37.80)	113 (49.30)		
	TT	17 (7.10)	13 (5.70)		
	C	357 (74.07)	319 (69.65)	2.267	0.132
	T	125 (25.93)	139 (30.35)		
Dominant model	CC	133 (55.20)	103 (45.00)	4.895	0.027
	CT + TT	108 (44.80)	126 (55.00)		
Recessive model	TT	17 (7.10)	13 (5.70)	0.373	0.542
	CT + CC	224 (92.90)	216 (94.30)		
Additive model	CC	133 (55.20)	103 (45.00)	0.001	0.974
	TT	17 (7.10)	13 (5.70)		
rs3813082	AA	193 (80.10)	180 (78.60)	0.651	0.722
	AC	42 (17.40)	45 (19.70)		
	CC	6 (2.50)	4 (1.70)		
	A	428 (88.80)	405 (88.43)	0.032	0.859
	C	54 (11.20)	53 (11.57)		
Dominant model	AA	193 (80.10)	180 (78.60)	0.157	0.692
	AC + CC	48 (19.90)	49 (21.40)		
Recessive model	CC	6 (2.50)	4 (1.70)	0.311	0.577^*^
	AC + AA	235 (97.50)	225 (98.30)		
Additive model	AA	193 (80.10)	180 (78.60)	0.266	0.753^*^
	CC	6 (2.50)	4 (1.70)		

SNP, single nucleotide polymorphism. ^*^: There is a frequency <5 at the identification point, and the *p*-value is calculated using a continuous corrected chi square test.

### 3.3 Analysis of Risk Factors for Coronary Heart Disease in Women

**Supplementary Table 5** outlines the classification of blood lipid parameters, SUA, and routine hematological indices. These classifications are based on the 2022 Chinese Clinical Blood Lipid Test Guide [17], the 2019 Basic Diagnosis and Treatment Guide for Gout and Hyperuricemia [18], and the ninth edition of the Diagnostic Standard [19]. These authoritative guidelines provide a standardized framework for assigning values and interpreting the diverse biochemical and hematological data in this study, ensuring consistency and reliability.

Spearman correlation analysis revealed that positive associations between CHD in women and multiple factors, including lifestyle factors (smoking and alcohol consumption), clinical conditions (hypertension and type 2 diabetes mellitus), and biochemical markers (BMI, TC, ApoB, Lp(a), WBC, NE, and PLT). The *rs2000813 CT* genotype also showed a positive correlation with CHD, whereas the *rs2000813 CC* genotype was inversely associated with CHD in women. Univariate logistic regression analysis identified hypertension, type 2 diabetes mellitus, advanced age, elevated BMI, and increased levels of TC, ApoB, Lp(a), WBC, NE, and PLT as risk factors for CHD in women, each with distinct ORs. Multivariate logistic regression further confirmed the following as significant predictors: hypertension (OR, 2.305; 95% confidence interval (CI), 1.438–3.696; *p* < 0.001), age ≥60 years (OR, 2.267; 95% CI, 1.392–3.692; *p* < 0.001), BMI ≥28 kg/m^2^ (OR, 8.634; 95% CI, 4.653–16.021; *p* < 0.001), TC ≥6.2 mmol/L (OR, 6.437; 95% CI, 1.074–38.577; *p* = 0.042), ApoB ≥1.1 g/L (OR, 2.504; 95% CI, 1.138–5.512; *p* = 0.023), and the *rs2000813 CT* genotype (OR, 1.614; 95% CI, 1.010–2.579; *p* = 0.045). These factors, with their respective ORs and 95% CIs, were significant predictors of CHD in women. Detailed statistical results are provided in Table 3.

**Table 3.** **Univariate and multivariate logistic regression analysis**.

Risk factor	One-way		Risk factor	Multi-factor	
	OR (95% CI)	*p*-value		OR (95% CI)	*p*-value
Hypertension	2.301 (1.590, 3.332)	<0.001	Hypertension	2.305 (1.438, 3.696)	<0.001
Diabetes	2.304 (1.474, 3.602)	<0.001	Diabetes	1.286 (0.749, 2.209)	0.362
Age ≥60 years	2.130 (1.440, 3.150)	<0.001	Age ≥60 years	2.267 (1.392, 3.692)	<0.001
BMI ≥28 kg/m^2^	4.438 (2.925, 6.735)	<0.001	BMI ≥28 kg/m^2^	8.634 (4.653, 16.021)	<0.001
TC ≥6.2 mmol/L	6.768 (1.966, 23.299)	<0.001	TC ≥6.2 mmol/L	6.437 (1.074, 38.577)	0.042
ApoB ≥1.1 g/L	2.461 (1.453, 4.168)	0.001	ApoB ≥1.1 g/L	2.504 (1.138, 5.512)	0.023
Lp(a) ≥300 mg/L	1.637 (1.045, 2.677)	0.032	Lp(a) ≥300 mg/L	1.422 (0.798, 2.534)	0.233
WBC >10 × 10^9^/L	4.409 (2.312, 8.408)	<0.001	WBC >10 × 10^9^/L	2.148 (0.303, 15.741)	0.438
2.0 × 10^9^/L ≤ NE < 7.0 × 10^9^/L	0.416 (0.258, 0.671)	<0.001	2.0 × 10^9^/L ≤ NE < 7.0 × 10^9^/L	1.102 (0.084, 14.544)	0.941
NE ≥7.0 × 10^9^/L	4.877 (2.668, 8.915)	<0.001	NE ≥7.0 × 10^9^/L	4.015 (0.214, 75.168)	0.352
PLT ≥300 × 10^9^/L	3.452 (2.186, 5.445)	<0.001	PLT ≥300 × 10^9^/L	3.064 (0.341, 27.560)	0.318
rs2000813CC	0.664 (0.460, 0.955)	0.027	rs2000813CC	-	0.082
rs2000813CT	1.606 (1.112, 2.318)	0.012	rs2000813CT	1.614 (1.010, 2.579)	0.045

BMI, body mass index; TC, total cholesterol; ApoB, apolipoprotein B; Lp(a), lipoprotein (a); WBC, white blood cell; NE, neutrophil count; PLT, platelet.

### 3.4 MDR-based Analysis of the Association Between Endothelial Lipase Gene-environment Interaction and the Development of Coronary Heart Disease in Women

This study suggests that the *rs2000813 CT* genotype may increase the risk of CHD in women, while the *rs3813082* locus of the endothelial lipase gene may influence lipid metabolism. To explore these genetic contributions further, we analyzed interactions between these loci and environmental factors—including smoking, alcohol consumption, hypertension, type 2 diabetes mellitus, age, BMI, and specific blood markers—using the MDR method. This approach constructed a multilevel interaction model integrating genetic and environmental variables, demonstrating strong predictive performance (**Supplementary Table 6**).

MDR analysis focused on interactions between the *rs2000813* and *rs3813082* loci of the endothelial lipase gene. **Supplementary Fig. 1A** illustrates risk-factor combinations associated with CHD in women. Dark-colored cells denote high-risk combinations, indicating increased CHD susceptibility, whereas light-colored cells represent low-risk combinations, suggesting reduced disease likelihood. White cells indicate no significant association with CHD. **Supplementary Fig. 1B**, highlights positive interactions between these loci in red, quantifying their individual main effects. Notably, *rs2000813* exhibited a stronger influence on CHD risk than *rs3813082*.

**Supplementary Fig. 2** presents the MDR analysis of interactions between the *rs2000813* and *rs3813082* loci of the *endothelial lipase* gene and environmental factors in in relation to CHD in women. Bar histograms distinguish the case group (left) from the control group (right), with dark cells indicating high-risk combinations, light cells representing low-risk combinations, and white cells denoting no significant association with CHD. This visualization elucidates the gene-environment interactions influencing CHD in women, offering insights into the complex interplay of genetic and environmental factors.

**Supplementary Fig. 3** depicts interaction tree highlighting patterns among risk factors for CHD in women. Traditional cardiovascular risk factors-including neutrophil count, BMI, platelet count, and WBC-cluster on a single main branch, exhibiting a strong negative interaction (shown in dark blue). The introduction of the *rs2000813* locus, alongside other risk factors such as alcohol consumption, age, smoking, lipoprotein(a), type 2 diabetes mellitus, hypertension, apolipoprotein B, and total cholesterol, attenuates this negative interaction, transitioning towards a positive interaction (indicated by green and yellow). Ultimately, a weak positive interaction emerges with *rs3813082*.

**Fig. 1.**
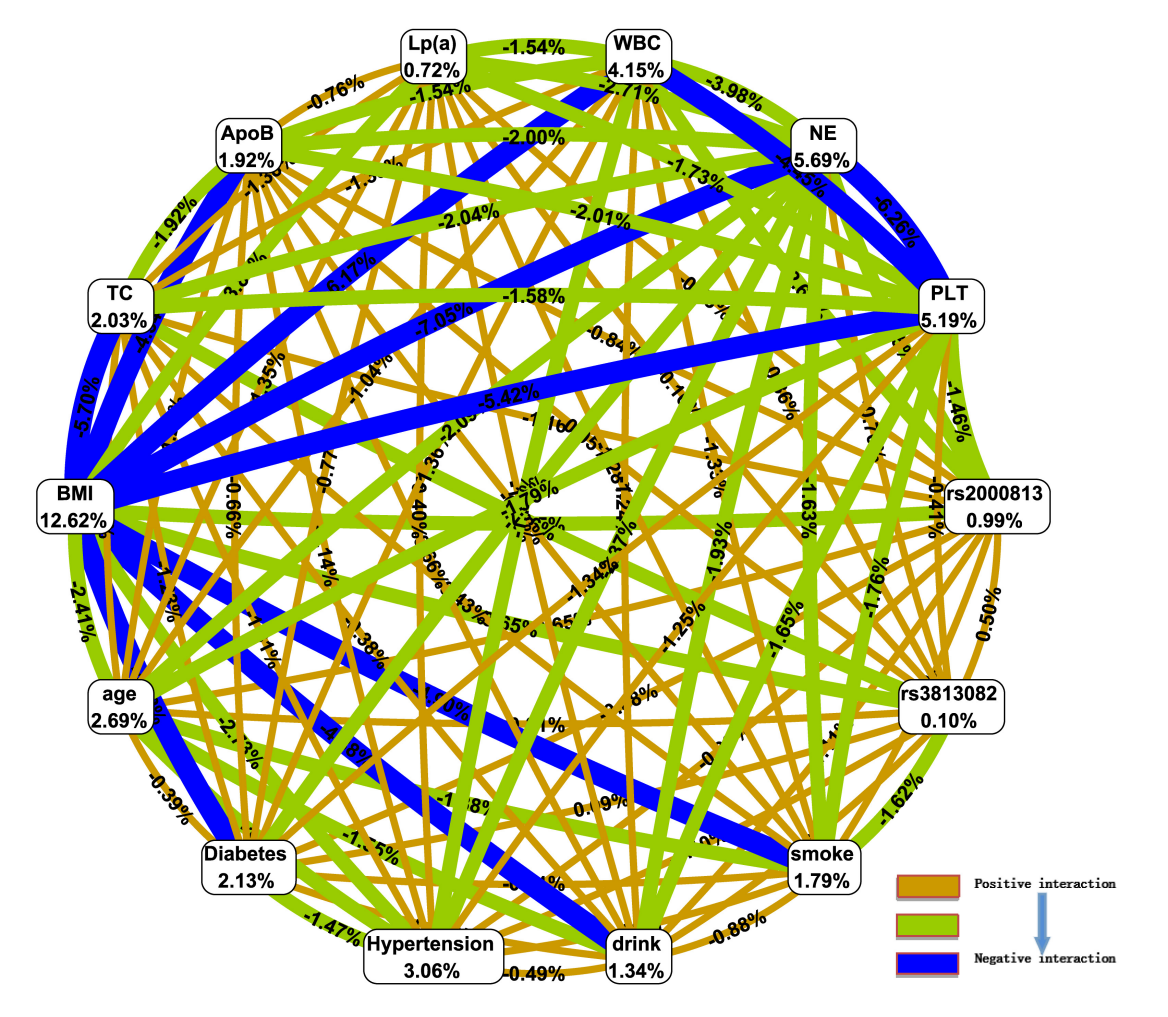
**Interaction ring network of *rs2000813* and *rs3813082* loci and gene-environment effects in the multifactor dimensionality reduction (MDR) model**. WBC, white blood cell; NE, neutrophil count; PLT, platelet; Lp(a), lipoprotein (a); ApoB, apolipoprotein B; TC, total cholesterol; BMI, body mass index.

In the interaction ring network of the MDR model (Fig. 1), node values represent the information gain from individual attributes (main effects), while values between nodes reflect the information gain from attribute pairs (interaction effects). The main effects of single attributes, ranked in descending order, are as follows: BMI, NE, PLT, WBC, hypertension, age, type 2 diabetes mellitus, TC, ApoB, smoking, alcohol consumption, *rs2000813*, Lp(a), and *rs3813082*. Among the interaction effects, the strongest positive interaction occurs between *rs2000813* and *rs3813082*, whereas the most pronounced negative interaction is observed between BMI and NE.

### 3.5 Construction and Validation of a Predictive Model for Coronary Heart Disease Risk in Women

**Fig. 2.**
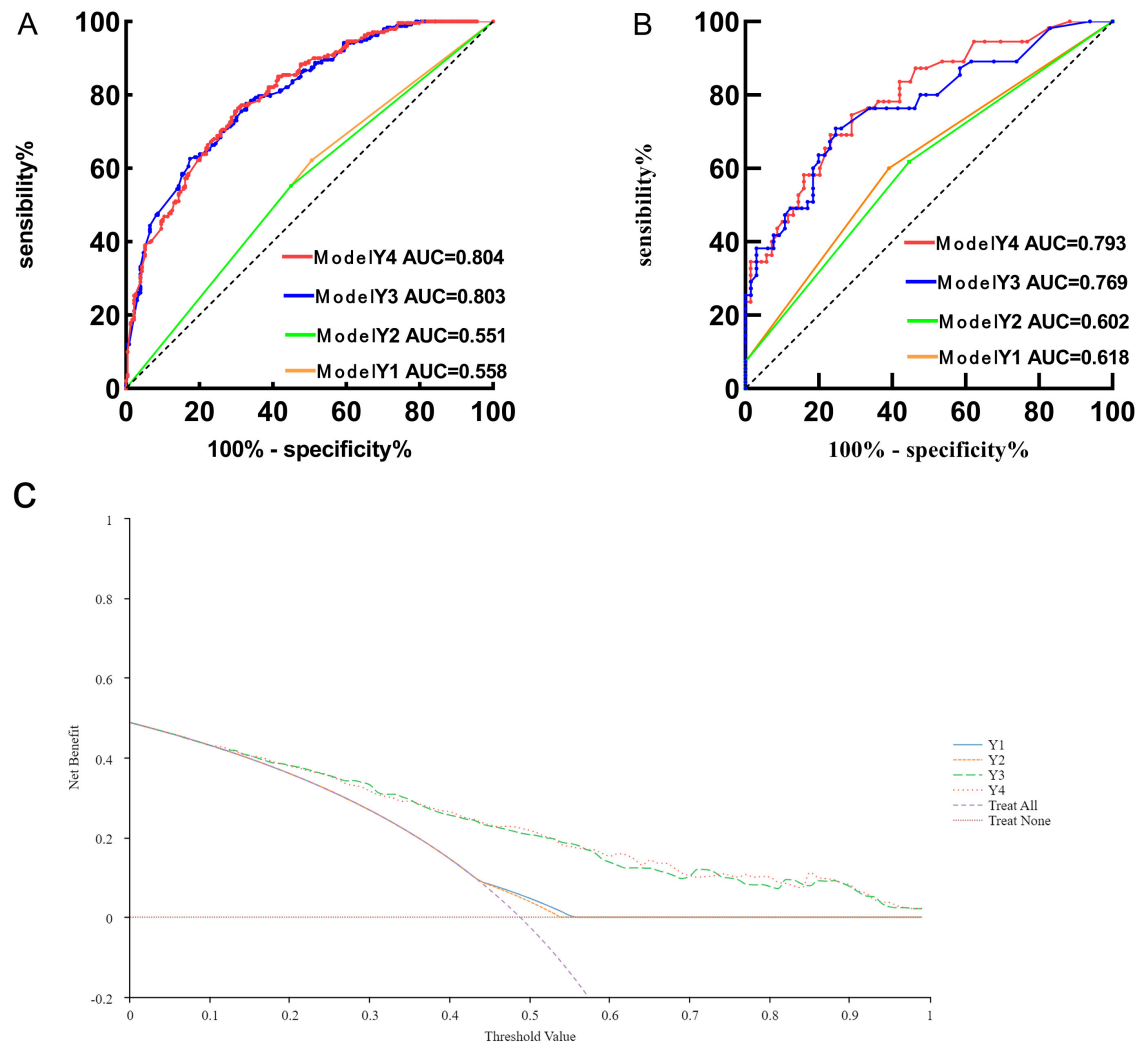
**Performance evaluation of coronary heart disease risk models in female patients — ROC curves for training (A) and validation (B) sets, and decision curves for training set (C)**. AUC, area under the curve; ROC, receiver operating characteristic.

Logistic regression analysis, conducted using SPSS software (version 26.0), generated prediction probabilities for four models. Model Y1 was defined by the *rs2000813 CT* genotype, while Model Y2 by the *rs2000813 CC* genotype. Model Y3 included traditional cardiovascular risk factors (e.g., smoking, hypertension, BMI), while Model Y4 combined all Model Y3 factors with both *rs2000813* genotypes. ROC curve analysis revealed that Model Y3 (risk ratio index [RRI] >0.344; sensitivity, 82.5%; specificity, 62.7%) and Model Y4 (RRI >0.515; sensitivity, 68.6%; specificity, 77.2%) outperformed Model Y1 and Y2 in predicting CHD risk in women. Model Y4 demonstrated strong predictive performance, achieving area under the curve (AUC) values of 0.804 (95% CI, 0.766–0.843; *p* < 0.001) and 0.793 (95% CI, 0.715–0.871; *p* < 0.001) in the training and validation sets, respectively. Decision curves for the female patient training set show Model Y4 yields high net benefit in predicting female coronary heart disease risk, demonstrating its good clinical applicability. AUC values and sensitivity/specificity metrics are detailed in Table 4 and Fig. 2.

**Table 4.** **Prediction value of model Y1, Y2, Y3 and Y4 for female coronary heart disease**.

Predictors	Youden’s index	Cut off point	Sensitivity (%)	Specificity (%)	AUC (95% CI)	*p*-value
Model Y1	0.115	0.495	0.493	0.622	0.558 (0.506, 0.610)	0.030
Model Y2	0.102	0.487	0.550	0.552	0.551 (0.499, 0.603)	0.056
Model Y3	0.452	0.344	0.825	0.627	0.803 (0.765, 0.842)	<0.01
Model Y4	0.458	0.515	0.686	0.772	0.804 (0.766, 0.843)	<0.01

AUC, area under the curve.

**Fig. 3.**
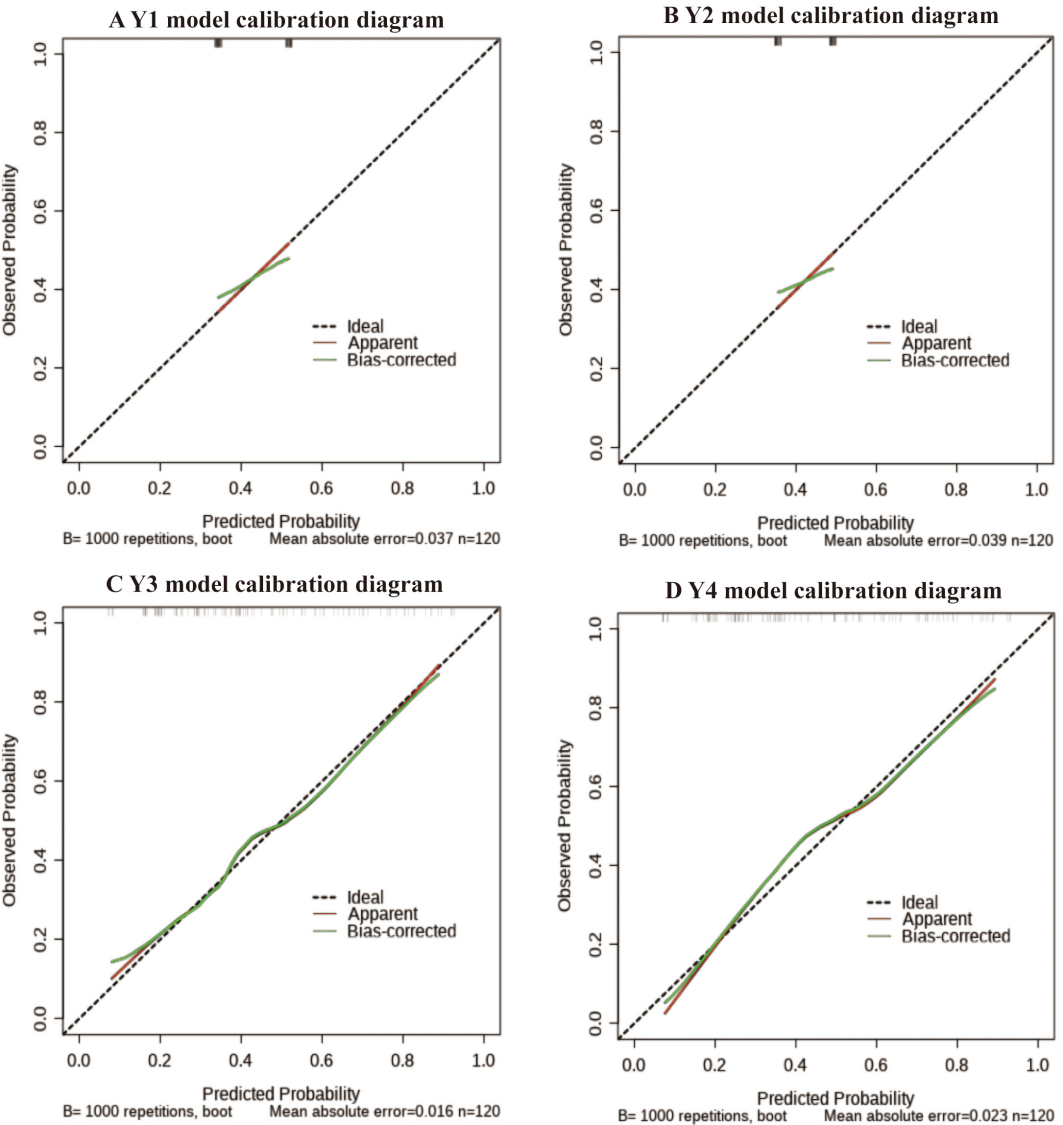
**Calibration diagram of female coronary heart disease risk model**. (A) Calibration curve of predicting CHD risk in women in the Model Y1. (B) Calibration curve of predicting CHD risk in women in the Model Y2. (C) Calibration curve of predicting CHD risk in women in the Model Y3. (D) Calibration curve of predicting CHD risk in women in the Model Y4. CHD, coronary heart disease. Calibration curve likely adhered closest to the 45-degree ideal line, indicating minimal deviation between predictions and actual outcomes.

In this study, 1800 participants were enrolled and divided into a modeling subset (1440 participants) and a validation subset (360 participants) in an 8:2 ratio. These models were reconstructed and validated in female patients. Of these, 590 were women, with 470 (229 CHD, 241 Controls) in the modeling subset and 120 (51 CHD, 69 Controls) in the validation subset. Statistical analysis confirmed no significant differences in the distribution of factors such as smoking status, hypertension prevalence, or *rs2000813* genotypes between the two subsets (*p* > 0.05), as detailed in Table 5. For predicting CHD risk in women, Model Y4 exhibited the highest predictive accuracy, as detailed in Table 4 and Fig. 3.

**Table 5.** **Comparison of the differences of the predicted influence factors among different groups of women**.

Items	All (n = 590)	Modeling subset (n = 470)	Validate subset (n = 120)	*p*-value
CHD	288 (48.1)	229 (48.7)	51 (42.5)	0.476
Smoking history	10 (1.7)	8 (1.7)	2 (1.7)	1.000
History of drinking	6 (1.0)	6 (1.3)	1 (0.8)	0.882
Hypertension	310 (52.5)	242 (51.5)	68 (56.7)	0.598
Diabetes	128 (21.7)	107 (22.8)	21 (17.5)	0.458
Age ≥60 years	395 (66.9)	313 (66.6)	82 (68.3)	0.937
BMI ≥28 kg/m^2^	193 (32.7)	158 (33.6)	35 (29.2)	0.650
TC ≥6.2 mmol/L	22 (3.7)	21 (4.5)	1 (0.8)	0.172
ApoB ≥1.1 g/L	95 (16.1)	73 (15.5)	22 (18.3)	0.758
Lp(a) ≥300 mg/L	110 (18.6)	88 (18.7)	22 (18.3)	0.995
WBC >10 × 10^9^/L	73 (12.4)	59 (12.6)	14 (11.7)	0.966
2.0 × 10^9^/L ≤ NE < 7.0 × 10^9^/L	478 (81.0)	379 (80.6)	99 (82.5)	0.898
NE ≥7.0 × 10^9^/L	88 (14.9)	71 (15.1)	17 (14.2)	0.967
PLT ≥300 × 10^9^/L	131 (22.2)	114 (24.3)	17 (14.2)	0.060
rs2000813CC	295 (50.0)	236 (50.2)	59 (49.2)	0.979
rs2000813CT	260 (44.1)	204 (43.4)	56 (46.7)	0.814

CHD, coronary heart disease; BMI, body mass index; TC, total cholesterol; ApoB, apolipoprotein B; Lp(a), lipoprotein (a); WBC, white blood cell; NE, neutrophil count; PLT, platelet.

## 4. Discussion

CAD is widely recognized for its genetic basis, with cardiovascular diseases (CVD) development driven by interactions between genetic and traditional risk factors. The association between gene polymorphisms and CHD is a prominent focus of global research due to its implications for understanding and managing CAD [20]. Investigating gene-environment interactions is essential not only for addressing the “missing heritability” of complex traits but also for elucidating the biological mechanisms underlying multifactorial diseases. This approach is central to genetics, providing deeper insights into how genetic and environmental factors jointly shape complex health conditions [21]. Genetic variants may not directly cause disease but can influence its onset and progression through subtle interactions with other genes or environmental exposures. This complexity underscores that genetic contributions to disease often manifest significantly only in combination with additional genetic or environmental factors [22]. Studying these interactions is critical for understanding disease etiology and the impact of environmental exposures on health outcomes. Focusing on clinical data and *EL* gene polymorphisms in CHD patients, this research aims to uncover associations between gene interactions and disease susceptibility. It also seeks to develop a risk prediction model to identify high-risk populations, offering valuable insights for targeted public health strategies and interventions.

Endothelial lipase, encoded by the *endothelial lipase* gene, is predominantly synthesized by vascular endothelial cells and exhibits robust phospholipase activity with minimal triglyceride lipase activity. This enzyme critically regulates serum HDL-C metabolism, influencing cholesterol transport and cardiovascular health [23]. HDL-C is widely acknowledged for its protective role against atherosclerosis, mediated by anti-inflammatory and antioxidant effects, inhibition of platelet aggregation, and promotion of re-endothelialization. Its association with the incidence and prognosis of CHD underscores its cardiovascular significance [24]. Initially, endothelial lipase was thought to contribute to CHD pathogenesis primarily through HDL-C regulation. Subsequent research, however, demonstrated additional roles, including facilitating macrophage adhesion to the vascular endothelium, promoting platelet aggregation and endothelial cell proliferation, and contributing to atherosclerosis. Notably, suppressing endothelial lipase expression during inflammation may reduce the severity of coronary atherosclerosis [25]. Among the triglyceride lipase family, endothelial lipase is considered the most influential in atherosclerosis, emphasizing its relevance to cardiovascular diseases research [26].

In this study, the *rs2000813 CT* genotype of the *endothelial lipase* gene was identified as a potential risk factor for CHD in women, whereas the *CC* genotype exhibited a protective effect. These findings align with prior study linking *endothelial lipase* gene single-nucleotide polymorphisms (SNPs) to increased CHD risk, reinforcing the role of genetic factors in this disease [27]. However, Elnaggar *et al*. [28] suggested that the *T* allele of the *rs2000813* (*584C/T*) variant may confer protection against CHD, implying a reduced risk for carriers. In contrast, Zhao *et al*. [29] reported that the CC genotype and C allele may predispose individuals to higher CHD risk, highlighting potential genetic contributions to disease susceptibility. The *rs2000813* genotype also appears linked to sex-specific differences in CHD incidence, potentially influenced by variations in sex hormone levels. Mehilli and Presbitero [30] underscored significant disparities in clinical presentation, complications, and cardiovascular risk profiles between men and women with CHD, emphasizing the need to consider sex-specific factors. Women’s susceptibility to endothelial dysfunction and occult CHD may stem from unique factors such as inflammation, mental stress, and autonomic or neuroendocrine dysregulation [31], contributing to sex-specific cardiovascular risks. The increased CHD risk in women with the *rs2000813 CT* genotype may reflect its specific effects on lipid metabolism and related pathways, though further research is required to elucidate the underlying mechanisms.

In this study, no significant association was observed between *rs3813082* polymorphism of the endothelial lipase gene and CHD (*p* > 0.05). This study did not identify an association between *ELrs3813082* gene polymorphism and coronary heart disease. However, it’s important to note that the *ELrs3813082* gene’s minimum allele frequency (MAF-*C* = 0.13) was very small in this study, and the sample size was relatively limited, which may have influenced the results. Larger studies are warranted to clarify the role of *rs3813082* in CHD.

This study employed multifactor dimensionality reduction analysis to examine gene-gene and gene-environment interactions in CHD. A positive interaction was identified between the *rs2000813* and *rs3813082* loci of the *endothelial lipase* gene. Among individual attributes, the main effects ranked in descending order of influence were BMI, NE, PLT, WBC, hypertension, age, type 2 diabetes mellitus, TC, ApoB, smoking, alcohol consumption, *rs2000813*, Lp(a), and *rs3813082*. The strongest interaction occurred between *rs2000813* and *rs3813082*.

Hartiala *et al*. [32] found that smoking attenuates the protective effect of the *ADAMTS7* gene on cardiovascular health, while increased physical activity amplifies the influence of three genetic loci on serum HDL-C levels and diminishes the effect of another locus. Air pollution, meanwhile, elevates cardiovascular disease risk by altering *CXCL12* expression, and X chromosome variants significantly regulate sex differences in cardiovascular disease incidence [32]. In this study, significant interactions were identified between the *rs2000813* and *rs3813082* loci of the endothelial lipase gene and environmental factors-including BMI, NE, PLT, WBC, hypertension, age, type 2 diabetes mellitus, TC, ApoB, smoking, alcohol consumption, and Lp(a)-collectively influencing CHD risk in women. These gene-environment interactions deepen our understanding of CHD pathogenesis and genetic susceptibility, offering critical insights for developing tailored lifestyle recommendations and therapeutic strategies.

Complex interactions between SNPs and traditional cardiovascular risk factors are pivotal in CHD pathogenesis. Integrating these factors with SNPs in CHD-related genes enhances our understanding of disease mechanisms. Notably, severe coronary atherosclerosis may develop asymptomatically for years before angina manifests [33]. Proactively identifying high-risk individuals and managing modifiable risk factors can substantially reduce adverse cardiac events [34]. Developing an effective risk prediction model based on clinical and laboratory data is essential for pinpointing high-risk populations, enabling targeted risk factor management, and optimizing healthcare resource allocation. For instance, Pattarabanjird *et al*. [35] combined the *TID3 rs11574* polymorphism with traditional risk factors, achieving 87.0% accuracy and an AUC of 0.840 in predicting CAD severity, markedly improving prediction efficiency. In this study, we integrated *endothelial lipase* gene polymorphisms (*rs2000813*, *rs3813082*) and traditional CHD risk factors (smoking, alcohol consumption, hypertension, type 2 diabetes mellitus, age ≥60 years, BMI ≥28 kg/m^2^, TC ≥6.2 mmol/L, ApoB ≥1.1 g/L, Lp(a) ≥300 mg/L, WBC >10 × 10^9^/L, 2.0 × 10^9^/L ≤ NE < 7.0 × 10^9^/L, NE ≥7.0 × 10^9^/L, PLT ≥300 × 10^9^/L) to predict CHD. This combined model yielded a sensitivity of 68.6%, specificity of 77.2%, and AUC of 0.804, demonstrating superior predictive performance compared to individual factors alone.

This study has several limitations. First, as a single-center study with a relatively small sample size, it may be subject to selection bias, potentially limiting the generalizability of the findings. Second, the prediction model underwent only internal validation, without external validation, necessitating further assessment of its clinical applicability and broader relevance. Consequently, future research should prioritize large-scale, multicenter, prospective studies to validate the predictive utility of *endothelial lipase* polymorphisms (*rs2000813* and *rs3813082*) combined with traditional cardiovascular risk factors for CHD incidence in women.

## 5. Conclusions

This study identified the *rs2000813 CT* genotype of the *endothelial lipase* gene as a potential risk factor for CHD in women. Furthermore, a synergistic interaction between *endothelial lipase* gene polymorphisms and environmental factors appears to influence CHD susceptibility in women. When integrated with traditional cardiovascular risk factors, this model exhibits robust predictive performance for CHD in women.

## Availability of Data and Materials

Data supporting the findings of this study are available from the corresponding author upon reasonable request within 1 year of publication of this article.

## Supplementary Material

Supplementary material associated with this article can be found, in the online version, at https://doi.org/10.31083/RCM37356.
