# Supplementary material for: Effect of Interactions Between Endothelial Lipase Gene Polymorphisms and Traditional Cardiovascular Risk Factors on Coronary Heart Disease Susceptibility
Source: Rev Cardiovasc Med. 2025 Jul 25;26(7):37356. doi: 10.31083/RCM37356 (PMC12326425; doi:10.31083/RCM37356)
Supplement: Supplementary file 1 [file 2153-8174-26-7-37356-s1.zip › Supplementary Figure.docx]

| B  A 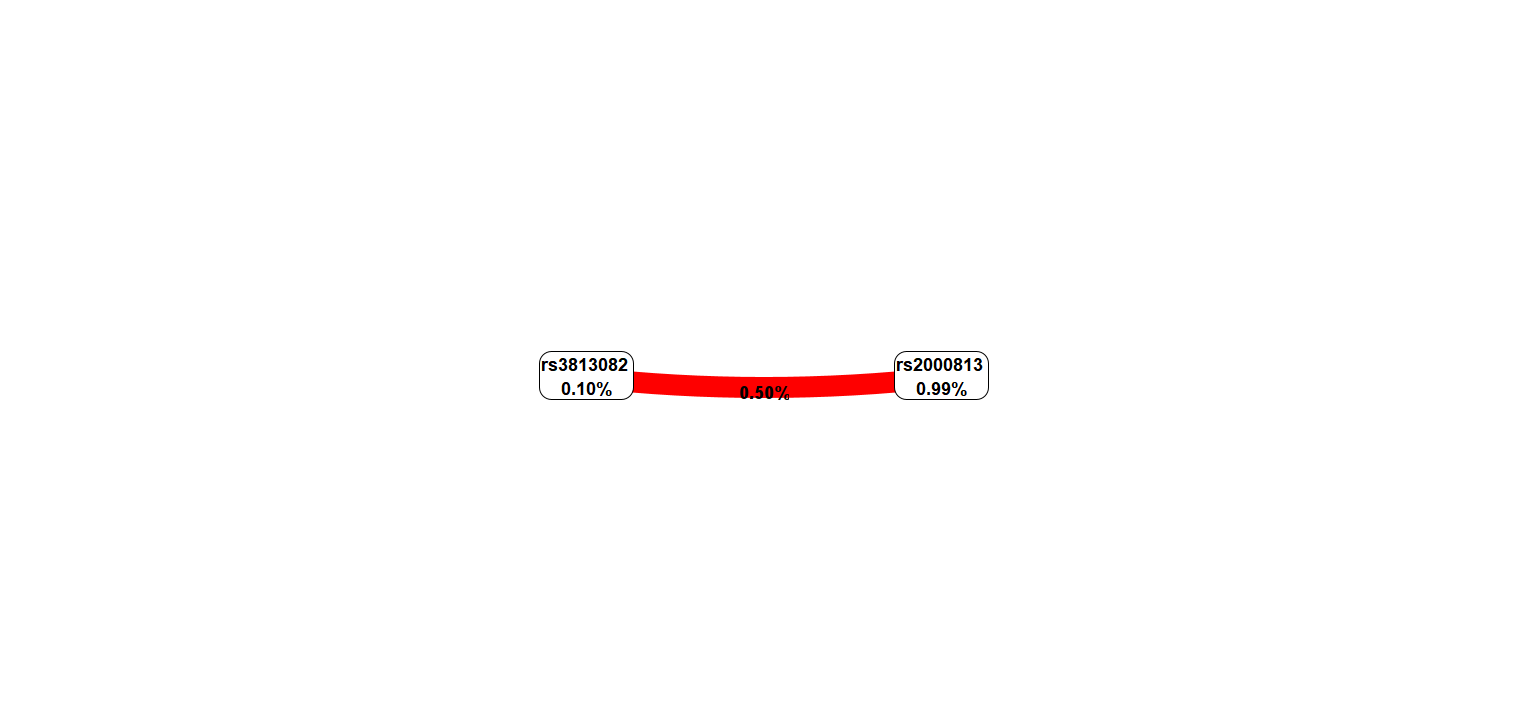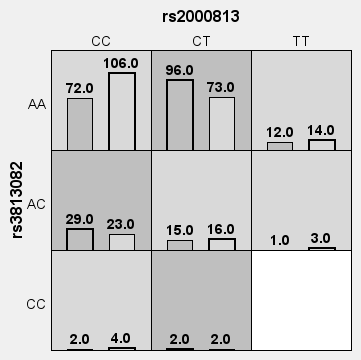 |
| --- |
| Supplementary Figure 1: Model of interaction between rs2000813, rs3813082 gene and gene MDR. |

| 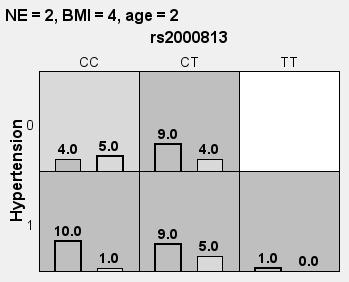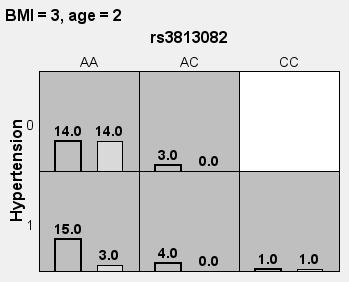 |
| --- |
| Supplementary Figure 2 : Interaction model of rs2000813, rs3813082 genes and environmental MDR. |

| 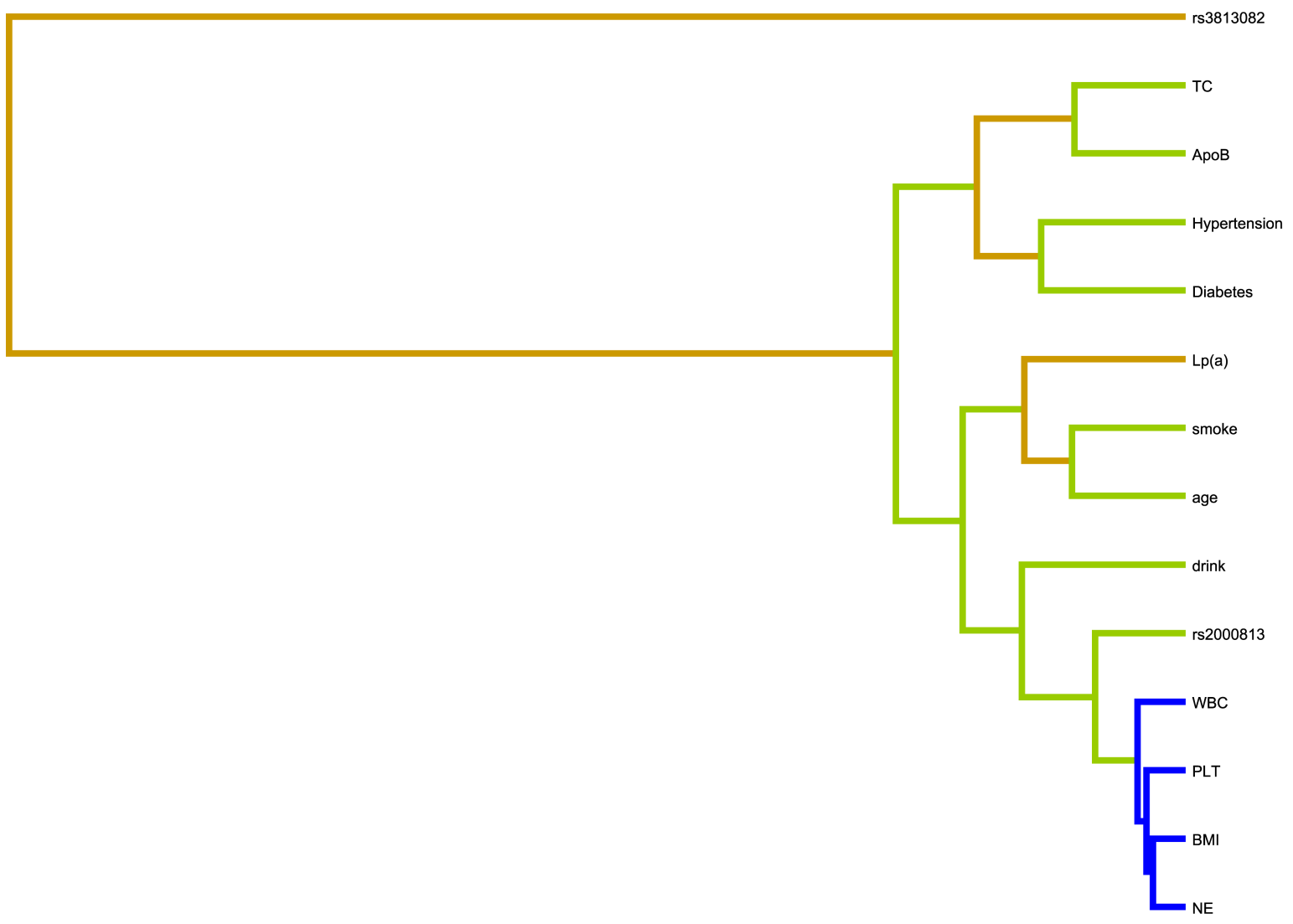 |
| --- |
| Supplementary Figure 3 : The dendrogram of the interaction between rs2000813, rs3813082 gene, and gene, and between gene and environment MDR. |
