# Supplementary material for: Effect of Interactions Between Endothelial Lipase Gene Polymorphisms and Traditional Cardiovascular Risk Factors on Coronary Heart Disease Susceptibility
Source: Rev Cardiovasc Med. 2025 Jul 25;26(7):37356. doi: 10.31083/RCM37356 (PMC12326425; doi:10.31083/RCM37356)
Supplement: Supplementary file 1 [file 2153-8174-26-7-37356-s1.zip › Supplementary Table.docx]

Supplementary Table 1: SNPs sites PCR primer design.

| SNP | Primer sequence (5’-3’) | Product size (bp) |
| --- | --- | --- |
| rs2000813 | F：AAACTCGTGTCAGCCCTGCAGAC  R：AAACTCGTGTCAGCCCTGCAGAT | 254 |
| rs3813082 | F：GACACCCAGAATCCCCTCTC  R：AGGAGGACAAAGGGGATGAC | 210 |

F is the forward primer and R is the reverse primer.

Supplementary Table 2: Hardy Weinberg balance test.

| SNP | Genotype | Control group (n=720) | | | Case group (n=720) | | |
| --- | --- | --- | --- | --- | --- | --- | --- |
|  |  | Actual value | Theoretical value | *p* | Actual value | Theoretical value | *p* |
| rs2000813 | CC | 381 (52.90) | 366 (50.83) | 0.186 | 351 (48.70) | 366 (50.83) | 0.713 |
|  | CT | 295 (41.00) | 310.5 (43.13) |  | 326 (45.30) | 310.5 (43.13) |  |
|  | TT | 44 (6.10) | 43.5 (6.04) |  | 43 (6.00) | 43.5 (6.04) |  |
| rs3813082 | AA | 534 (74.20) | 543 (75.42) | 0.972 | 552 (76.70) | 543 (75.42) | 0.304 |
|  | AC | 172 (23.90) | 163.5 (22.71) |  | 155 (21.50) | 163.5 (22.71) |  |
|  | CC | 14 (1.90) | 13.5 (1.87) |  | 13 (1.80) | 13.5 (1.87) |  |

Supplementary Table 3: Risk analysis of coronary heart disease among different genotypes.

| SNP | Genotype | Control group (n=720) | Case group (n=720) | *x^2^* | *p* |
| --- | --- | --- | --- | --- | --- |
| rs2000813 | CC | 381 (52.90) | 351 (48.70) | 2.789 | 0.248 |
|  | CT | 295 (41.00) | 326 (45.30) |  |  |
|  | TT | 44 (6.10) | 43 (6.00) |  |  |
|  | C | 1057 (73.40) | 1028 (71.40) | 1.461 | 0.267 |
|  | T | 383 (26.60) | 412 (28.60) |  |  |
| Dominant model | CC | 381 (52.90) | 351 (48.70) | 2.501 | 0.114 |
|  | CT+TT | 339 (47.10) | 369 (51.30) |  |  |
| Recessive model | TT | 44 (6.10) | 43 (6.00) | 0.012 | 0.912 |
|  | CT+CC | 676 (93.90) | 677 (94.00) |  |  |
| Additive model | CC | 381 (52.90) | 351 (48.70) | 0.068 | 0.795 |
|  | TT | 44 (6.10) | 43 (6.00) |  |  |
| rs3813082 | AA | 534 (74.20) | 552 (76.70) | 1.219 | 0.544 |
|  | AC | 172 (23.90) | 155 (21.50) |  |  |
|  | CC | 14 (1.90) | 13 (1.80) |  |  |
|  | A | 1240 (86.10) | 1259 (87.40) | 1.092 | 0.296 |
|  | C | 200 (13.90) | 181 (12.60) |  |  |
| Dominant model | AA | 534 (74.20) | 552 (76.70) | 1.214 | 0.271 |
|  | AC+CC | 186 (25.80) | 168 (23.30) |  |  |
| Recessive model | CC | 14 (1.90) | 13 (1.80) | 0.038 | 0.846 |
|  | AC+AA | 706 (98.10) | 707 (98.20) |  |  |
| Additive model | AA | 534 (74.20) | 552 (76.70) | 0.076 | 0.783 |
|  | CC | 14 (1.90) | 13 (1.80) |  |  |

Supplementary Table 4: Risk analysis of coronary heart disease among different genotypes in male.

| SNP | Genotype | Control group (n=479) | Case group (n=491) | *x^2^* | *p* |
| --- | --- | --- | --- | --- | --- |
| rs2000813 | CC | 248 (51.80) | 248 (50.50) | 0.204 | 0.903 |
|  | CT | 204 (42.60) | 213 (43.40) |  |  |
|  | TT | 27 (5.60) | 30 (6.10) |  |  |
|  | C | 700 (73.07) | 709 (72.20) | 0.184 | 0.668 |
|  | T | 258 (26.93) | 273 (27.80) |  |  |
| Dominant model | CC | 248 (51.80) | 248 (50.50) | 0.155 | 0.693 |
|  | CT+TT | 231 (48.20) | 243 (49.50) |  |  |
| Recessive model | TT | 27 (5.60) | 30 (6.10) | 0.098 | 0.754 |
|  | CT+CC | 452 (94.40) | 461 (93.90) |  |  |
| Additive model | CC | 248 (51.80) | 248 (50.50) | 0.142 | 0.707 |
|  | TT | 27 (5.60) | 30 (6.10) |  |  |
| rs3813082 | AA | 341 (71.20) | 372 (75.80) | 2.925 | 0.232 |
|  | AC | 130 (27.10) | 110 (22.40) |  |  |
|  | CC | 8 (1.70) | 9 (1.80) |  |  |
|  | A | 812 (84.76) | 854 (86.97) | 1.945 | 0.163 |
|  | C | 146 (15.24) | 128 (13.03) |  |  |
| Dominant model | AA | 341 (71.20) | 372 (75.80) | 2.604 | 0.107 |
|  | AC+CC | 138 (28.80) | 119 (24.20) |  |  |
| Recessive model | CC | 8 (1.70) | 9 (1.80) | 0.037 | 0.847 |
|  | AC+AA | 471 (98.30) | 482 (98.20) |  |  |
| Additive model | AA | 341 (71.20) | 372 (75.80) | 0.04 | 0.950 |
|  | CC | 8 (1.70) | 9 (1.80) |  |  |

Supplementary Table 5: Assignment table of each variable.

| Identifier | Variable | Assignment | Identifier | Variable | Assignment |
| --- | --- | --- | --- | --- | --- |
| Y | CHD | 0=No，1=Yes | X11 | LDL-C≥4.1mmol/L | 0=No，1=Yes |
| X1 | Gender | 1=Male，2=Female | X12 | ApoA (g/L) | 1=“<1.2”  2=“≥1.2&<1.6”  3=“≥1.6” |
| X2 | Smoking history | 0=No，1=Yes | X13 | ApoB (g/L) | 1=“<0.8”  2=“≥0.8&<1.1”  3=“≥1.1” |
| X3 | History of drinking | 0=No，1=Yes | X14 | Lp(a)≥300 mg/L | 0=No，1=Yes |
| X4 | Hypertension | 0=No，1=Yes | X15 | SUA≥420 μmol/L | 0=No，1=Yes |
| X5 | Diabetes | 0=No，1=Yes | X16 | SCR (μmol/L) | 1=“<40”  2=“≥40&<120”  3=“≥120” |
| X6 | Age (years) | 1=“<45”  2=“≥45&<60”  3=“≥60” | X17 | WBC (10^9^/L) | 1=“<4”  2=“≥4&<10”  3=“≥10” |
| X7 | BMI (kg/m^2^) | 1=“<18.5”  2=“≥18.5&<24”  3=“≥24.0&<28.0”  4=“≥28.0” | X18 | NE (10^9^/L) | 1=“<2.0”  2=“≥2.0&<7.0”  3=“≥7.0” |
| X8 | TG≥2.3 mmol/L | 0=No，1=Yes | X19 | PLT (10^9^/L) | 1=“<100”  2=“≥100&<300”  3=“≥300” |
| X9 | TC≥6.2 mmol/L | 0=No，1=Yes | X20 | rs2000813 | 1=CC,2=CT,3=TT |
| X10 | HDL-C<1.0 mmol/L | 0=No，1=Yes | X21 | rs3813082 | 1=AA,2=AC,3=CC |

BMI: Body mass index; TG: Triglycerides; TC: Total cholesterol; HDL-C: High-density lipoprotein cholesterol; LDL-C: Low-density Lipoprotein cholesterol; ApoA: Apolipoprotein A; ApoB: Apolipoprotein B; Lp(a): Lipoprotein a; SUA: Blood uric acid; Scr:serum creatinine; WBC: White blood cell count; NE: Neutrophil count; PLT: Platelet count.

Supplementary Table 6: Interaction model of rs2000813, rs3813082 gene and traditional cardiovascular risk factors MDR.

| Interaction model | Training Bal.Acc. CV | Testing Bal.Acc. CV | CV Consistency | *P* |
| --- | --- | --- | --- | --- |
| BMI | 0.6622 | 0.6231 | 10/10 | <0.001 |
| Hypertension, BMI | 0.7097 | 0.6891 | 5/10 | <0.001 |
| Age, BMI, NE | 0.7341 | 0.7110 | 9/10 | <0.001 |
| rs3813082, Hypertension, Age, BMI | 0.7493 | 0.6573 | 4/10 | <0.001 |
| rs2000813, Hypertension, Age, BMI, NE | 0.7781 | 0.6502 | 4/10 | <0.001 |
| rs2000813, Hypertension, Age, BMI, ApoB, PLT | 0.8129 | 0.6453 | 10/10 | <0.001 |
| rs2000813, Hypertension, Age, BMI, ApoB, Lp(a), PLT | 0.8432 | 0.6058 | 5/10 | <0.001 |
| rs2000813,rs3813082, Hypertension, Diabetes, Age, BMI, ApoB, PLT | 0.8679 | 0.5791 | 5/10 | <0.001 |
| rs2000813,rs3813082, Hypertension, Diabetes, Age, BMI, ApoB, Lp(a), PLT | 0.8880 | 0.5669 | 8/10 | <0.001 |
| rs2000813,rs3813082, Hypertension, Diabetes, Age, BMI, ApoB, Lp(a), WBC, PLT | 0.9028 | 0.5763 | 10/10 | <0.001 |
| rs2000813, rs3813082, Smoking, Hypertension, Diabetes, Age, BMI, ApoB, Lp(a), WBC, PLT | 0.9072 | 0.5805 | 9/10 | <0.001 |
| rs2000813,rs3813082, Smoking, Hypertension, Diabetes, Age, BMI, TC, ApoB, Lp(a), WBC, PLT | 0.9091 | 0.5827 | 9/10 | <0.001 |
| rs2000813,rs3813082, Smoking, Drinking, Hypertension, Diabetes, Age, BMI, TC, ApoB, Lp(a), WBC, PLT | 0.9091 | 0.5827 | 10/10 | <0.001 |
| rs2000813,rs3813082, Smoking, Drink, Hypertension, Diabetes, Age, BMI, TC, ApoB, Lp(a), WBC, NE, PLT | 0.9091 | 0.5827 | 10/10 | <0.001 |

BMI: Body mass index; TC: Total cholesterol; ApoB: Apolipoprotein B; Lp(a): Lipoprotein A; WBC: White blood cell count; NE: Neutrophil count; PLT: Platelet count.
